# Subset-Specific Expression of Toll-Like Receptors by Bovine Afferent Lymph Dendritic Cells

**DOI:** 10.3389/fvets.2017.00044

**Published:** 2017-04-03

**Authors:** Dirk Werling, Jayne C. Hope, Nazneen Siddiqui, Stephanie Widdison, Chris Russell, Paul Sopp, Tracey J. Coffey

**Affiliations:** ^1^The Royal Veterinary College, Hatfield, Hertfordshire, UK; ^2^Institute for Animal Health, Newbury, Berkshire, UK

**Keywords:** toll-like receptors, dendritic cell, afferent lymph, dendritic cell subsets, bovine

## Abstract

Within the ruminant system, several possibilities exist to generate dendritic cells migrating out from the tissue into the regional draining lymph nodes as afferent lymph dendritic cells (ALDCs). Here, we analyzed toll-like receptor (TLR) 1–10 mRNA expression by using quantitative real-time PCR in highly purified subsets of bovine ALDC. As TLR expression may be influenced by pathogens or vaccines and their adjuvant, it is necessary to understand what TLRs are expressed in a steady-state system to elucidate specific differences and to potentially optimize targeted vaccines. In this study, we have assessed the TLR expression profiles of the four main bovine ALDC subsets [cDC1 and cDC2 (subsets 2–4)]. We demonstrate differences in TLR expression between the four subsets that may reflect the ability of these cells to respond to different pathogens or to respond to adjuvants.

## Introduction

Dendritic cells (DC) play a crucial role in the immune response; they are the key antigen-presenting cell (APC) orchestrating adaptive immune responses and are particularly important in effecting potent T-cell responses. Peripheral DC act as sentinels and, upon antigen recognition, migrate from the site of infection in afferent lymphatic vessels to lymph nodes. This is associated with a process of maturation enabling DC to interact with T-cells and B-cells to shape the ensuing adaptive immune response. It is well established that DC exist as heterogenic subsets with divergent phenotypes and functions (1).

In order to access DC draining from the periphery in afferent lymphatic vessels, a surgical cannulation model has been used (2). This has allowed detailed characterization of DC in ruminants, pigs, rats, and mice (3–6). In the bovine system, cannulation of skin-draining pseudo-afferent lymphatic vessels (after surgical removal of the prescapular lymph node) has enabled the detailed characterization of subsets of *ex vivo* bovine afferent lymph DC (ALDC) (1, 2, 7).

Initial analyses revealed that there were two major subpopulations of ALDC (1, 8), with the major subpopulation expressing the signal regulatory protein-α (CD172a) and low or no expression of the integrin CD11a, and the minor population not expressing CD172a, but showing high levels of CD11a expression. These subpopulations of ALDC were shown to differ in their ability to stimulate T-cells in order to affect tolerance or infection control (1). Subsequent studies showed these two populations differ in their cytokine expression profile as well as their ability to stimulate T-cells (9, 10). These two populations, both shown to express high levels of endocytic receptor CD205 (DEC-205) (11), have been further defined based predominantly on expression of the markers CD1b, CD5, CD21, CD13, CD26, and the mannose receptor (CD206), in addition to CD172a and CD11a. These studies suggest that the previously defined CD172a^+^ and CD172a^−^ ALDC represent classical (c)DC1 (CD11a^+^, CD13^+^, CD26^+^, CD172a^low/−^), and cDC2 subsets (CD11a^−^, CD13^−^, CD26^−^, CD172a^+^). These subsets are similar, but distinctively different from the recently described porcine cDC1 and cDC2 subsets (12). Within the bovine cDC2 subset, three major subpopulations have been defined: CD172a^+^CD206^+^CD1b^++^CD21^+^, CD172a^+^CD206^−^CD1b^+^CD21^+/−^, and CD172a^+^CD206^−^CD1b^+/−^CD21^−^ (1, 8, 11, 13, 14).

In addition to phenotypical differences within these smaller subsets, there is evidence for differential function including the capacity to uptake antigen, and cytokine secretion [(1, 9, 14) and Presentation S2 in Supplementary Material]. These data suggested that the subsets within the CD172a^+^ cDC2 population were maturation dependent subsets.

Understanding the functional properties of skin draining ALDC is important as these are potential targets for vaccines and adjuvants delivered into the skin. Recent evidence suggests that bovine ALDC subsets interact differentially with vaccines (14–16). Phagocytosis of the attenuated vaccine strain *Mycobacterium bovis* bacillus Calmette–Guerin (BCG) was shown, both *in vitro* and *in vivo*, to be predominantly associated with the CD172a^+^CD206^+^CD1b^++^ (cDC2_2) subset of ALDC, which was subsequently shown to induce higher expression of interferon (IFN)γ by CD4^+^ lymphocytes (14–16). Similar experiments with a human adenovirus (AdV5) expressing Ag85A, a secreted mycobacterial protein thought to be a promising candidate as a protective antigen, found that the CD172^+^ (cDC2) population cultured with AdV5-85A induced significantly higher IFNγ expression than either CD172^+^ (cDC2) cells cultured with AdV5 alone or CD172a^−^ (cDC1) cells cultured with AdV5-85A (14–17). This raises interesting questions regarding the potential of these ALDC subsets to recognize different microbial-associated molecular patterns (MAMPs).

The toll-like receptors (TLRs) play a critical role in the mammalian innate immune response. These pathogen recognition receptors recognize conserved MAMPs as well as endogenous danger associated molecular patterns, thereby inducing the release of pro-inflammatory cytokines (18). As in humans, there are 10 known TLRs in cattle (19, 20) with each recognizing a different MAMP. The ligand binding repertoire of several TLRs is expanded through both homo- and hetero-dimerization, particularly in those belonging to the TLR1 family, TLR1, TLR2, TLR6, and TLR10 (21). TLRs are expressed by APC, and in humans, their expression has been shown to vary significantly between APC subsets (22–25).

In a previous study, we demonstrated that differences exist between the TLR expression profiles of bovine monocytes, monocyte-derived macrophages, alveolar macrophages, monocyte-derived DC, bone marrow-derived DC, and CD172a^+^ (cDC2) and CD172a^−^ (cDC1) subpopulations of ALDC (20). However, to date, quantitative measurement of TLR expression by specific subsets of bovine ALDC has not been performed. As TLR expression may be influenced by pathogens or vaccines and their adjuvant, it is necessary to understand what TLRs are expressed in a steady-state system to elucidate specific differences and to potentially optimize targeted vaccines. In this study, we have assessed the TLR expression profiles of four bovine ALDC subsets: CD172a^−^ (cDC1; population 1) and three subsets of cDC2 (populations 2–4): CD172a^+^CD206^+^CD1b^++^ (cDC2_2), CD172a^+^CD206^−^CD1b^+^ (cDC2_3), and CD172a^+^CD206^−^CD1b^+/−^ (cDC2_4). We demonstrate differences in *TLR* expression between the four subsets that may reflect the ability of these cells to respond to different pathogens or adjuvants.

## Materials and Methods

### Cannulation of Pseudo-Afferent Lymphatic Ducts and Isolation of ALDC Subsets

Three Holstein Friesian calves were used for cannulation performed essentially as previously described (2, 7). In brief, pseudo-afferent lymph draining the skin was collected into sterile plastic bottles containing heparin (10 U mL^−1^), penicillin, and streptomycin with bottles replaced every 8–12 h. All experiments conformed to local and national guidelines on the use of experimental animals and had been approved by the Ethics Committee at the Institute for Animal Health under Home Office project license PPL 30/2327.

Lymph cells were analyzed using a FACSCalibur (Becton Dickinson, Oxford, UK) and FCS Express software (*DeNovo* Software, Los Angeles, CA, USA). Expression of cell surface molecules was assessed using directly labeled monoclonal antibodies (mAb; AbD Serotec, Oxford, UK) or biotinylated mAb measured using fluorescently labeled streptavidin. Isotype and concentration matched anti-avian mAb were used as controls (26). ALDC were isolated from lymph based on their size (large forward scatter) and high intensity expression of CD205 (11, 27). Expression of CD1b and CD172a was measured using the mouse anti-bovine mAb CC14 and CC149, respectively (1, 28, 29). CD206 expression was determined using the anti-human mAb 3.29B1 (Beckman Coulter, Inc., High Wycombe, UK), coupled to phycoerythrin, previously shown to cross-react with bovine CD206 (30). Subsets of ALDC were purified to >99% purity using a FACSAria cell sorter (Becton Dickinson), and the overall gating/sorting strategy is shown in Presentation S1 in Supplementary Material.

### RNA Extraction and cDNA Preparation for Real-time TaqMan^®^ PCR

RNA was isolated using the RNeasy mini kit (Qiagen, Hilden, Germany) according to the manufacturer’s instructions. The quality and quantity of RNA were assessed using the NanoDrop spectrophotometer (NanoDrop products, Wilmington, DE, USA). RNA was treated using DNA-*free*™ (Ambion, Austin, TX, USA) to remove contaminating genomic DNA. Fifty nanograms of RNA were used to produce cDNA using the Superscript III reverse transcription kit (Invitrogen Ltd., Refrenshew, UK). cDNA was treated using RNase H (Invitrogen Ltd.) to remove complementary RNA prior to cDNA quantification using the NanoDrop spectrophotometer.

### Real-time TaqMan^®^ PCR for Quantification of TLR cDNA

Primers and probes for bovine *TLR1* and *TLR6* have previously been described (31). Primers and probes (Table 1) were synthesized by Sigma-Genosys Ltd. (Haverhill, UK) and Eurogentec Ltd. (Romsey, UK), with at least one primer or probe designed to span an intron–exon boundary where possible. Probes were labeled at the 5′ end with the reporter dye FAM (6-carboxyfluorescein) and at the 3′ end with the quencher dye TAMRA (6-carboxytetramethyl-rhodamine). Quantitative PCR was carried out using TaqMan^®^ FAST Universal PCR Mastermix (Applied Biosystems, Warrington, UK) on the ABI Prism 7500 Fast Real-Time PCR System (Applied Biosystems), with 100 ng cDNA as a starting template. The amplification program consisted of an initial denaturation step of 95°C for 20 s, followed by 40 cycles of 95°C for 3 s, and 60°C for 30 s. Samples were tested in triplicate, and results were quantified by comparison with plasmid standard curves containing known copy numbers of cloned full-length target genes.

**Table 1:** **Primers and probes used for bovine toll-like receptor (TLR) mRNA quantification**.

Gene	Forward primer 5′–3′	Reverse primer 5′–3′	Probe 5′–3′
Bo*TLR1*	GCACCACAGTGAGTCTGGAA	GTACGCCAAACCAACTGGAG	TGTGTGCTTGATAATGGGTGTCCT
Bo*TLR2*	ACGACGCCTTCGTGTCCTAC	GCTCCTGGACCATGAGGTTC	CGAGCGGGATTCCTACTGGGTGG
Bo*TLR3*	AAAGAGTTCTCTCCTGGGTGTT	TGCTCAGGGACAGATTCTCA	CAATGCCAAGCTGAGCCCCA
Bo*TLR4*	TGGAGGACATGCCAGTGCT	CACCGACACTGATGATCGT	AGTTTCAGGAACGCCACTTGTCAGCTG
Bo*TLR5*	CTAGACCTGGGTGGAAGTCAG	AGGGATGAAGGTAAAGACTCTGAA	TTCCTGTGGTCTCTCCGATGCTG
Bo*TLR6*	CCTGCCCATCTGTAAGGAAT	TAGGTGCAAGTGAGCAATGG	TTGGCAACTTGACCCAACTGAATTTC
Bo*TLR7*	GCTGAAGACTGTCCCTGAGA	TTTGAGCTGAGGTCCAGATG	TCCAACTGTTCCCGCAGCCTC
Bo*TLR8*	TCCACATTTGAAACGAAGACC	ACATCGGTCAGTCTGGGAAC	CCTGACGTTCAGATTTCTGTCCATC
Bo*TLR9*	CACCATCTTCAACGACCTGA	CTTCTCCAGGGACACCAGAC	TCCTTCGCCCACCTGCACCT
Bo*TLR10*	TTTCTTTGTGGCGGAGTTC	AAAAGTCAGCCAGCCAGATT	ACAAACCCATTTTCCCAGCCTCC

cDNA samples were analyzed for gene expression. Samples from three or four time points were included per animal representing a time-period of 7–19 days post-cannulation; there were no significant differences in gene expression over the cannulation period (data not shown). Relative expression values were calculated using the “Relative Quantitation of Gene Expression Experimental Design and Analysis: Relative Standard Curve Method” (Applied Biosystems Technical Bulletin: “Guide to Performing Relative Quantitation of Gene Expression Using Real-Time Quantitative PCR”). In summary, the gene expression levels for each *TLR* were normalized to the expression level of the normalizer gene, representing the large fragment of the RNA polymerase (RPLPO), based on published data (32).

### Statistical Analysis

Statistical analyses of data were carried out using Microsoft^®^ Excel 2002 (Microsoft Co., Redmond, WA, USA) and GraphPad Prism 5.01 for Windows (GraphPad Software, San Diego, CA, USA, www.graphpad.com). After assessing data for normal distribution, differences between ALDC subsets were assessed by repeated measures two-way analysis of variance followed by Bonferroni *t*-tests.

## Results and Discussion

The availability of antibodies to cell surface expressed molecules and transcriptome analysis has facilitated the more precise definition of cDC1 and cDC2 subsets in bovine afferent lymph. Transcriptomics analysis has enabled the comparative analysis of DC subsets across species demonstrating that there are conserved subsets (33), which differ in cytokine expression and function. However, a detailed transcriptome analysis has not been performed for bovine ALDC, and specifically the expression profile of TLRs on these cells has not been described. Differential expression of TLRs (and other pattern recognition receptors) likely influences the capacity of DC subsets to respond to pathogens, vaccines, and adjuvants.

Distinct subsets of bovine DC have been described draining the skin, which have differential capacities to respond to vaccines (14–16) and to interact with T lymphocytes (1). Whether these subsets also express different profiles of pattern recognition receptors such as TLR was investigated herein.

Bovine ALDC defined by the expression of CD172a, CD206, and CD1b were purified by FACS sorting into four subpopulations as described previously (14). These populations were defined as CD172a^−^ (cDC1, population 1), and subsets within the cDC2 equivalents, namely, CD172a^+^CD206^+^CD1b^++^ (population 2; cDC2_2), CD172a^+^CD206^−^CD1b^+^ (population 3; cDC2_3), and CD172a^+^CD206^−^CD1b^+/−^ (population 4; cDC2_4). These four identified subsets of ALDC were analyzed for their expression levels of all 10 known bovine TLR genes (*TLR1–10*) (19, 20). The cDC1 subset represents a minor fraction of ALDC draining the skin of the neck/head region and, compared to the cDC2 subset, has been shown to be less capable of inducing responses in T lymphocytes (1). The cDC2 subset also interacts less efficiently with vaccines and appears to play a minor role in immune response induction in lymph nodes draining the skin.

**Figure 1:**
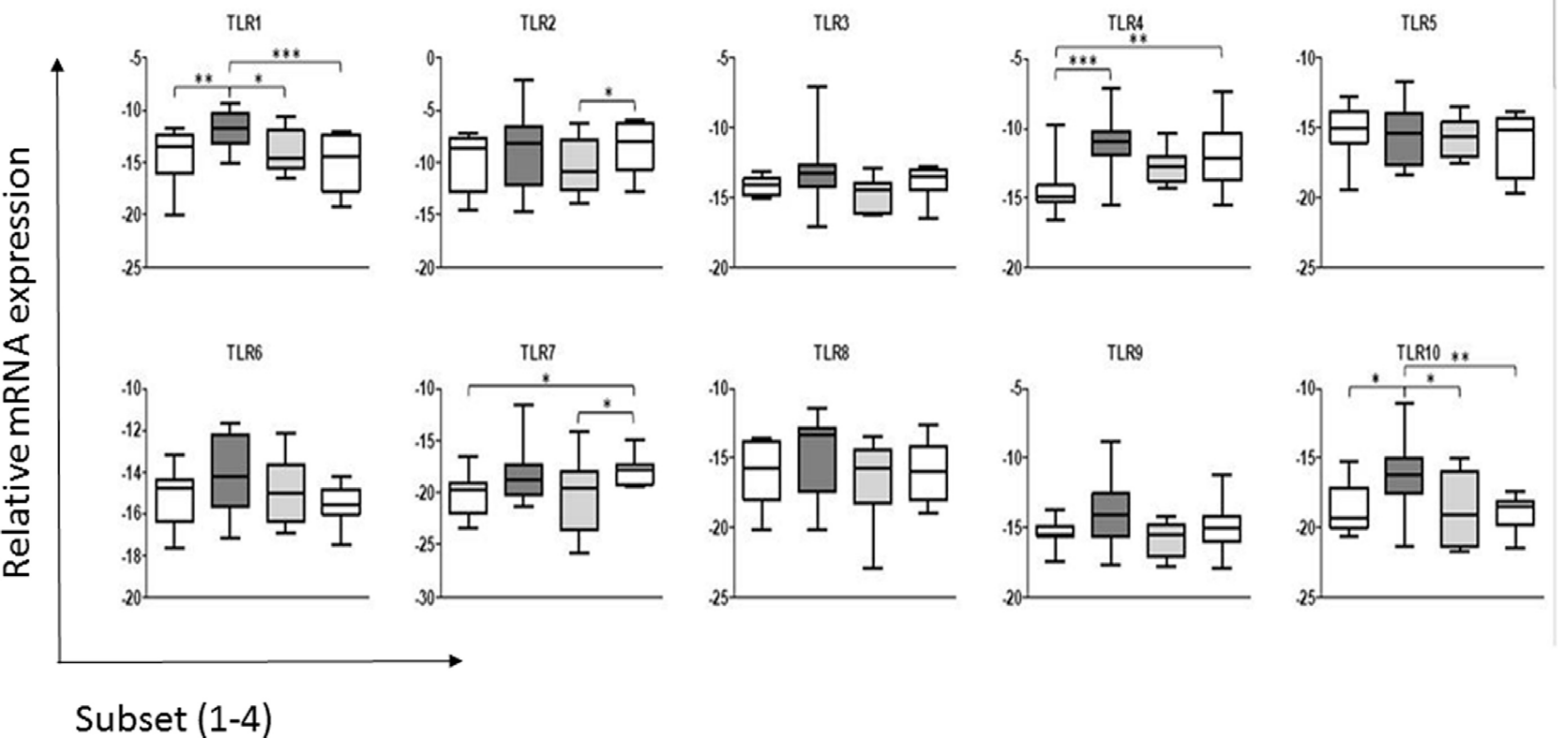
**Expression of toll-like receptors (*TLRs*) by the subsets of dendritic cells (DC) in bovine afferent lymph**. Subsets of afferent lymph dendritic cells (ALDCs), as used in this study (see Presentation S2 in Supplementary Material), were analyzed by real-time PCR for their comparative expression of the 10 bovine *TLRs*. Expression of each *TLR* was normalized to the expression level of the housekeeping gene representing the large fragment of the RNA polymerase (RPLPO). Differences in expression between the ALDC subsets were assessed by repeated measures two-way analysis of variance followed by Bonferroni *t*-tests. Significant differences in expression were detected for *TLR1, TLR2, TLR4, TLR7*, and *TLR10*. Asterisks denote significant differences in mRNA expression of a given TLR between subsets: ***p value <0.001, **p value <0.01, *p value <0.05.

The results (Figure 1) indicated that under steady-state conditions each of the ALDC subsets expressed mRNA for all 10 TLR genes. In general and similar to what was reported with regards to the shift in the cDC2 subset associated with the loss of CD206 and CD1b, the more mature cell subsets (cDC2_3 and cDC2_4; Presentation S2 in Supplementary Material) appeared to have reduced TLR mRNA expression. Thus, the more immature ALDC express higher levels of TLRs in line with their major role in antigen recognition, compared to roles for the more mature subsets in stimulating T-cells and priming of an adaptive immune response. It has been shown that only the more immature subset of ALDC, which expresses high level of CD206 (herein described as cDC2_2), is able to phagocytose *M. bovis* BCG and its virulent parent strain *M. bovis* (14). Since this subset shows the highest level of *TLR* expression overall, this may potentially explain the differential recognition and subsequent uptake of BCG (14). Interestingly, a recent study of *Mycobacterium tuberculosis* highlighted strain-specific TLR recognition, with the potential to influence the ensuing immune response thereby contributing to the outcome of infection (34).

Analysis of the expression levels of each TLR gene found that *TLR2* was expressed more highly across all four ALDC subsets compared to the other TLR genes. This is in line with data assessing the surface expression of TLR2 (35), which was shown to be similar in both the CD172a^−^ (cDC1) and CD172a^+^ (cDC2) subsets. This suggests that mRNA expression corresponds to surface expression of TLRs on ALDC, at least for this molecule (35). Interestingly, differences in mRNA levels for *TLR2* were detected between cDC2_3 and cDC2_4, but the functional relevance of these is not known.

When comparing ALDC subsets, there were no differences in expression of *TLR3, TLR5, TLR6, TLR8*, or *TLR9*. Interestingly, as described previously for the human CD141^+^ mDC subset (36), we found that the cCD2_2 subset expressed *TLR1* and *TLR10*, a TLR absent in mice but present in the rat (37), at higher levels than the other subsets. TLR10 belongs to the TLR2 subfamily, together with TLR1, TLR2, and TLR6, though the ligand specificity of this TLR in most mammals remains unknown. The implication of the strong expression of *TLR10* on the function of cDC2_2 ALDC remains to be determined.

Significant differences were seen in the expression of some TLR genes between the DC subsets. The levels of *TLR4* were significantly lower in the cDC1 (CD172a^−^) subset compared to the cDC2_2 and cDC2_4 subsets. Finally, *TLR7* expression was significantly higher in the cCD2_4 subset compared to cDC1 and cDC2_3 populations.

These data differ from those reported previously where the total CD172a^+^ (cDC2) ALDC were compared to the CD172a^−^ (cDC1) subset (20). The previous study used semiquantitative assessment of TLR expression and identified CD172a^+^ (cDC2) ALDC as expressing higher levels of *TLR3, TLR7*, and *TLR9*, showing a trend toward higher *TLR5* expression (20). Although we identified increased levels of *TLR7* in the CD172a^+^ (cDC2) subsets compared to the CD172a^−^ (cDC1) subset, we were unable to identify increases in *TLR3, TLR5*, or *TLR9*. This likely reflects the differences in sensitivity of the qPCR used herein compared to semiquantitative PCR used previously and the separation of the total cDC2 population into its subpopulations.

The TLR expression repertoire of each subset may determine their ability to respond to pathogens. The increased levels of *TLR1* and *TLR10* in the cDC2_2 subset compared to the others may indicate an increased responsiveness to triacylated bacterial lipopeptides, which represent the TLR1/TLR2 heterodimer ligands (38). The increased levels of these two TLRs in this subset may also reflect the maturation stage of the ALDC, as expression of CD206 is associated with immature DC, which is specialized for antigen recognition (14, 39). TLR4 was expressed at a lower level in the cDC1 population compared to two of the cDC2 populations, CDC2_2 and cDC2_4. As this TLR recognizes bacterial LPS (40), it suggests that the cDC1 subset may not effectively recognize Gram-negative bacterial species.

In conclusion, we have shown that ALDC subsets present a restricted pattern of *TLR* expression, with major differences seen in *TLR1, TLR4, TLR7*, and *TLR10*. The role of these rare subsets in the bovine immune response, and whether the differences in TLR mRNA expression highlighted in this study relate to functional differences between the subsets, remains to be determined. It is interesting to speculate why these subsets of migrating cells express different levels of *TLRs*, particularly if one assumes that migration only occurs in response to a pathogenic stimulus. One possibility is that MAMPs or whole pathogens, which have been able to enter the lymphatic ducts, will be recognized by ALDC subsets, either driving T-cell polarization or fine-tuning the T-cell response (41). Indeed, *Salmonella* was found to travel free in lymph or associated with cells, largely with lymph monocytes and granulocytes but less with DC, and induced a strong influx of these phagocytic cells in afferent lymph (42). Our hypothesis could be further supported by the fact that cDC1 ALDC expressed significantly lower levels of *TLR1, TLR4*, and *TLR10* and have been shown to be poor at antigen presentation. Alternatively, DC may migrate constitutively within the lymph as part of homeostatic surveillance, and their TLR expression profiles reflect those of DCs resident within the skin in the steady state. The understanding of the contribution of each DC subset to a physiological immune response is particularly relevant for rational vaccine design.

## Author Contributions

DW, TJC and JH planned and designed experiments, and, together with SW, wrote the manuscript. PS sorted cells. SW, NS and CR performed experiments and helped with writing the manuscript.

## Conflict of Interest Statement

The authors declare that the research was conducted in the absence of any commercial or financial relationships that could be construed as a potential conflict of interest.

## Supplementary Material

Presentation S1 **Sort gates used to purify afferent lymph dendritic cell (ALDC) subpopulations**. Lymph was stained with mAb to CD205, CD172a, CD206, and CD1b as described in Section “Materials and Methods.” **(a)** The first gate set (Gate 1) was to identify large forward scatter cells. **(b)** These gated cells were assessed for expression of CD205 and CD172a. Two major populations of ALDC were further gated: Gate 2 CD205^+^CD172a^−^ and Gate 3 CD205^+^CD172a^+^. The CD205^+^CD172a^−^ (Gate 2) cells were sorted and are referred to as cDC1 in the text and figures. **(c)** CD205^+^CD172a^+^ (Gate 3) cells were further assessed for expression of CD206 and CD1b. Cells expressing CD206 and CD1b (Gate 4; cDC2_2), expressing high level CD1b but not CD206 (Gate 5, cDC2_3) and moderate to low levels of CD1b but not CD206 (Gate 6, cDC2_4) were sorted.

Presentation S2 **Phenotypical and functional characterization of bovine afferent lymph cell subsets**. Expression of surface antigens on cDC1 and cDC2 subsets is shown. Acetylcholine esterase positive (Ach+) afferent lymph dendritic cells are depicted, potentially suggesting different maturational stages of the same population. High levels of expression (^++^), low levels of expression (^+/−^), and the presence of expressing and non-expressing cells (^+/−^) are indicated for some antigens. Functional abilities of these subsets to stimulate T-cells are shown underneath the diagram.
