# Supplementary figures and images for: Subset-Specific Expression of Toll-Like Receptors by Bovine Afferent Lymph Dendritic Cells
Source: Front Vet Sci. 2017 Apr 3;4:44. doi: 10.3389/fvets.2017.00044 (PMC5376590; doi:10.3389/fvets.2017.00044)

## Slide 1
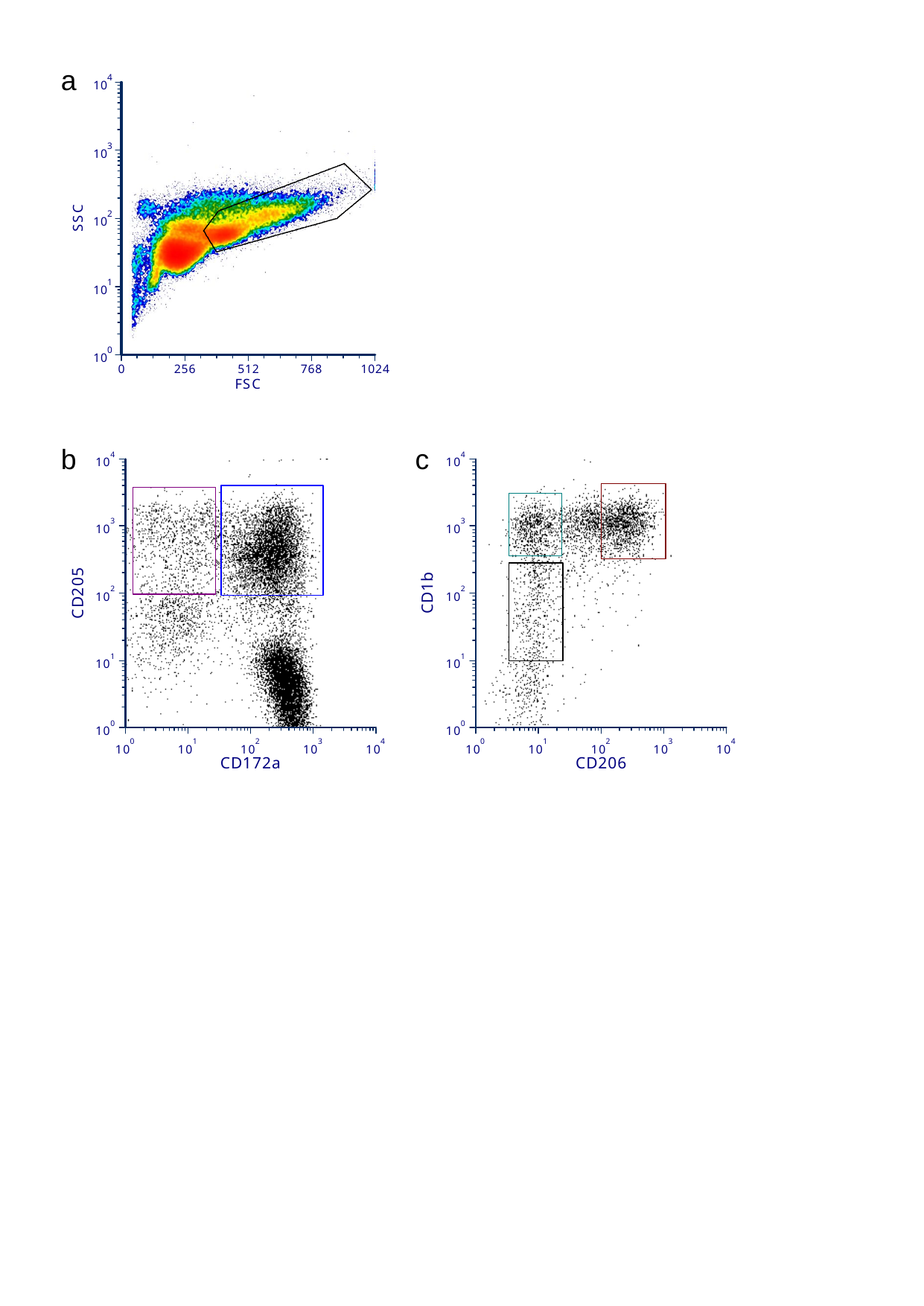

a
b
c

Supplement: Presentation S1 — Sort gates used to purify afferent lymph dendritic cell (ALDC) subpopulations. Lymph was stained with mAb to CD205, CD172a, CD206, and CD1b as described in Section “Materials and Methods.” (a) The first gate set (Gate 1) was to identify large forward scatter cells. (b) These gated cells were assessed for expression of CD205 and CD172a. Two major populations of ALDC were further gated: Gate 2 CD205+CD172a− and Gate 3 CD205+CD172a+. The CD205+CD172a− (Gate 2) cells were sorted and are referred to as cDC1 in the text and figures. (c) CD205+CD172a+ (Gate 3) cells were further assessed for expression of CD206 and CD1b. Cells expressing CD206 and CD1b (Gate 4; cDC2_2), expressing high level CD1b but not CD206 (Gate 5, cDC2_3) and moderate to low levels of CD1b but not CD206 (Gate 6, cDC2_4) were sorted. [file Presentation_1.PPTX]

## Slide 1
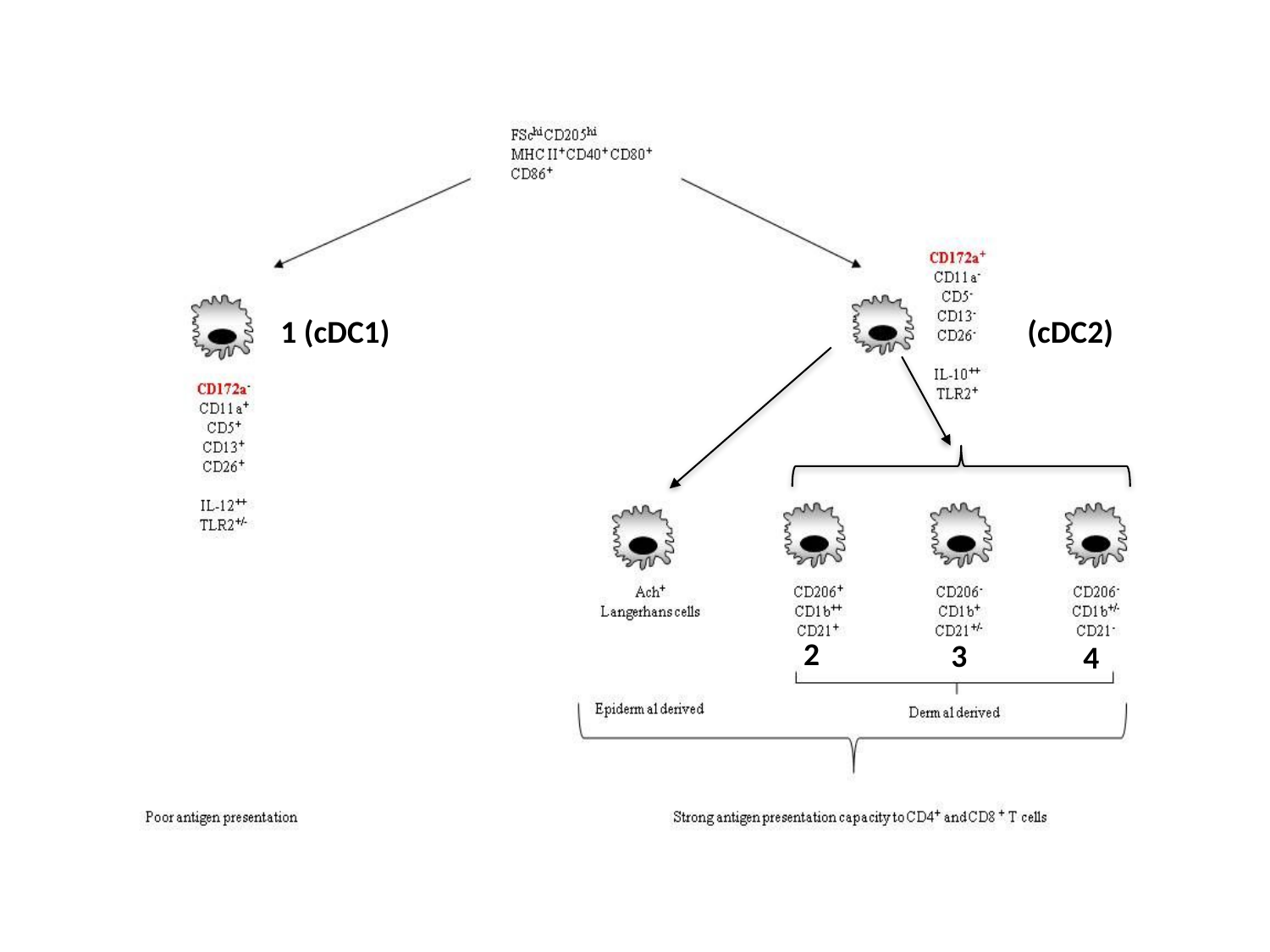

1 (cDC1)
(cDC2)
2
3
4

Supplement: Presentation S2 — Phenotypical and functional characterization of bovine afferent lymph cell subsets. Expression of surface antigens on cDC1 and cDC2 subsets is shown. Acetylcholine esterase positive (Ach+) afferent lymph dendritic cells are depicted, potentially suggesting different maturational stages of the same population. High levels of expression (++), low levels of expression (+/−), and the presence of expressing and non-expressing cells (+/−) are indicated for some antigens. Functional abilities of these subsets to stimulate T-cells are shown underneath the diagram. [file Presentation_2.PPTX]
